# A case study on the application evaluation of integrative medical service model for the improvement of quality of life for dementia patients and caregivers

**DOI:** 10.1097/MD.0000000000032282

**Published:** 2022-12-23

**Authors:** Moon Joo Cheong, Do-Eun Lee, Jeesu Kim, Il-Hong Son, Sung Chul Kim, Hyung Won Kang

**Affiliations:** a Rare Diseases Integrative Treatment Research Institute, Wonkwang University Jangheung Integrative Medical Hospital, Jangheung-gun, Jeollanam-do, Republic of Korea; b Department of Korean Neuropsychiatry Medicine, College of Korean Medicine, Wonkwang University, Iksan-si, Jeollabuk-do, Republic of Korea; c Department of Neurology, Wonkwang University Sanbon Hospital, Gunpo-si, Gyeonggi-do, Republic of Korea; d Department of Acupuncture & Moxibustion Medicine, Gwangju Korean Medical Hospital of Wonkwang University, Nam-gu, Gwangju, Republic of Korea.

**Keywords:** caregiver, dementia, integrative medical service model, quality of life

## Abstract

Since 4 major diseases and geriatric diseases require consistent management, individuals with any of these diseases cannot live alone and need caregivers’ assistance. Given these characteristics, an integrative medical service model for 4 major diseases and geriatric diseases was developed in Korea, currently. Dementia, one of the typical geriatric diseases, requires caregivers’ assistance from the beginning because of its enormous burden. Thus, it is necessary to provide an integrative medical service that can improve the quality of life (QoL) for both patients and caregivers. Therefore, this study aims to collect various feedback by applying an integrative medical service, which was developed to improve the QoL in patients with dementia and their caregivers, to a single case, and to modify and improve the integrative medical service model based on the results.

**Method/design:**

The integrative medical service program, which was developed to improve the QoL in patients with dementia and their caregivers in Korea, will be used for a patient-caregiver pair. This is an observational study with quantitative and qualitative feedback from various viewpoints. The program will be conducted in 8 sessions (twice a week, within 120 minutes). The patient will receive both Western and Korean medicine, and an integrative service will be provided to improve cognitive rehabilitation and QoL. Feedback collected at each session will be reflected on the program of the subsequent session.

**Results:**

This study will then modify and improve the program with feedback and provide integrative medical services to a patient with dementia and caregiver.

**Discussion:**

Patients with dementia need a program that would help them maintain cognitive function, and caregivers need a program that would improve their QoL by reducing the caregiving burden. This study is unique because the developed program is performed after modification based on feedback from the previous session. Accordingly, the patient and caregiver can check which program is the most satisfactory and helpful in improving their QoL. We expect that this study can modify the integrative medical service model to the optimized patient-based model. This study can also be used as basic data for a clinical pathway development study that applies the modified model to medical institutes.

## 1. Introduction

Integrative medicine is a patient-focused, whole-person medical practice that manages and treats patients, including physical and psychiatric aspects and all aspects of lifestyle.^[1]^ It has been proposed as a solution to improve side effects, complications, and dysfunction that appear in the course of the treatment for major and geriatric diseases by accepting all Western, Korean, and complementary and alternative medicines.^[2]^ Recently, an integrative medical service model for the 4 major diseases (cardiac and cerebrovascular diseases, cancer, and rare incurable diseases) and geriatric diseases has been developed in Korea.^[3]^ The targeted 4 major diseases and geriatric diseases have high prevalence and recurrence rates even after complete recovery and require continuous management.^[4]^ Patients with diseases require caregivers’ assistance because they cannot live independently. An integrative medical service model developed based on these features provides whole-person psychosocial interventions to improve patients and caregivers’ quality of life (QoL) and facilitate social participation.^[3]^

Globally, >55 million people are living with dementia, which is one of the typical geriatric diseases, and it is the 7th leading cause of death.^[5]^ According to 2018 data from Statistics Korea, the incidence of dementia was the second highest number among hospitalized patients aged ≥65 years.^[6]^ In Korea, the number of patients with dementia caused by injury or diseases tripled from 2010 to 2019, showing that the rate of increase in the number of patients with dementia was much faster than the rate of increase in the number of the elderly population.^[7]^ Accordingly, national attention to dementia has increased and the Korean government has promoted the National Responsibility for Dementia System since 2017.^[8]^ The National Responsibility for Dementia System reduces the burden of medical care expenses on the patients with dementia and provides long-term care services, but service is mainly provided from a provider’s point of view.^[7]^ Also, since dementia is characterized by loss of cognitive functioning and abnormal behavior-related symptoms, early provision of caregiver’s care is required.^[9]^ However, current medical service is overlooking caregivers’ management.^[10]^ Therefore, a “patient- and caregiver-centered integrative medical service” that encompasses not only patients with dementia but also their caregivers is required. Accordingly, this study aims to apply the integrative medical service model to 1 caregiver-patient pair and proposed a protocol to conduct it. This single-case study aims to collect the patient and caregiver’s opinion and to modify and improve the integrative medical service model based on the collected opinion. This can increase the accessibility of the integrative medical service model to the field and modify the model as higher reliability.

## 2. Method/design

### 2.1. Methodology

This is an observational study to collect the status of the actual application of the integrative medical service program, which was developed in Korea to improve patients with dementia and their caregivers’ QoL. Quantitative and qualitative feedback from diversified perspectives will be obtained to facilitate the application and refinement of the program in practical settings by researcher and clinical research coordinator. In order to obtain research consent, the research manager will explain the procedure to the participants in the research progress, compensation, and withdrawal. In addition, collect, share and maintain personal information about potential and registered participants. In order to protect confidentiality before, during and after trial, the Research Officer will store all data on the Research Officer’s personal computer, act in accordance with the management of the Agency Institutional Review Board (IRB), and fulfill his responsibilities. All data will be discarded 3 years after the end of the study.

### 2.2. Study participant

The study will include 1 dementia patient visiting an outpatient clinic of the study institute and his/her caregiver as participants. According to the study by Boddy CR,^[11]^ the sample size in a case study will be determined depending on the situation and can be different per scientific paradigm conducting a partial investigation. Based on this theory, a total of 2 participants will be selected. Expert sampling among purposive sampling will be used to select samples for investigation. Specific inclusion and exclusion criteria are as follows:

#### 2.2.1. Patient.

Inclusion criteria:

1. Male and female adults aged between 20 years and 85 years.
2. Among the patients treated with Western and Korean medicine, a patient clinically diagnosed with major neurocognitive disorder 1 year or before according to the Diagnostic and Statistical Manual of Mental Disorders, Fifth Edition. (Those with frontotemporal lobar degeneration, Lewy body disease, traumatic brain injury, substance and drug use disorders, human immunodeficiency virus infection, prion diseases, or Huntington disease are an exception.
3. Whose mini-mental state examination (MMSE) score is between 18 and 23 at the time of screening.
4. Can read the questionnaire and answer the questions.
5. Understand the purpose of this study and agree to participate in the study via a written form.

Exclusion criteria:

1. Has a history of psychotic symptoms, such as schizophrenia spectrum, delusional disorder, bipolar disorder, alcohol, or substance abuse disorders, diagnosed with Diagnostic and Statistical Manual of Mental Disorders, Fifth Edition.
2. Has chronic diseases that may affect the study results.
3. Has participated in another clinical trial within the past month.
4. Is unable to communicate clearly due to dementia or mild cognitive impairment (MCI).
5. Is unable to communicate and has difficulty in reading and completing the questionnaire.
6. Is unable to conduct the study due to the patient’s medical condition at the discretion of the principal investigator or study staff.

#### 2.2.2. One designated caregiver.

Inclusion criteria:

1. Designated adult caregiver (aged between 20 and 65 years) of the selected patient; has voluntarily agreed to participate in this study (a caregiver is a reliable adult who takes care of family or family’s daily activities).

Exclusion criteria:

1. Is unable to conduct the study at the discretion of the principal investigator or study staff because the individual has a communication disorder due to a serious psychiatric problem, intellectual disability, or mood disorder.

### 2.3. Study procedures

#### 2.3.1. The integrative medical service model.

An integrative medical service model for post-stroke cognitive impairment will be used. Since this is a cognitive impairment-centered model, we deemed that it can be applied to dementia, a major neurocognitive disorder.

The developed model is described in detail below.

First, patients visiting a medical institute can receive medical service as a diagnosis and treatment via cooperative/integrative Western and Korean medicine, the features of Korean medicine, and will receive optional complementary and alternative medicine as an integrative service. For patients with cognitive impairment, an online/offline integrative service will be provided to maintain cognitive function by using exercise techniques and cognitive games. Assessment will be conducted after providing service to create improvements. When the patient returned home, he/she will receive follow-up support from a general coordinator and integrative medical team.

Second, an integrative medical service will be provided to not only the patients but also to caregivers. Specifically, caregivers will be provided with information about financial support to actually receive support by connecting with the community. Moreover, family members who are exhausted due to long-term care will be provided with counseling and psychotherapy for mental and physical support.

This is a prospective observational study that receives feedback on revisions by applying the “treatment performance” step, which provides complementary and alternative medicine as an integrative service. The study includes medical treatment in both Western and Korean medicine among the developed integrative medical service model, for the patient with dementia and caregiver.

#### 2.3.2. Integrative medical service program.

##### 2.3.2.1. Composition of the program

The integrative medical service program, an optional complementary and alternative medicine service, is described below.

The program was developed based on the integrative medical service model for cognitive rehabilitation and QoL improvements in patients with post-stroke cognitive impairment, which was previously developed as a part of the integrative medical support program of the Ministry of Health and Welfare. The composition of the program will be decided upon consultation with the medical staff so that its application to the field is easy when conducting an application study based on this model. Also, feedback from every session will be considered to improve the program. An individual who is previously treated with Western and Korean medicine will be included as a participant, and medical services provided for 8 sessions in which the program applied will be the same throughout the study. The program is described in detail below.

Medical service:

1) The patient will be treated via care pathways in existing Western and Korean medicine for dementia patients, and drug and acupuncture treatments.

Integrative service:

1) Patient program: The patient will take breathing meditation, cognitive rehabilitation, and exercise programs.

- Breathing meditation will be performed using a 5-minute guided or recorded video to control one’s breath.
- Cognitive rehabilitation and exercise programs would help the patient maintain cognitive function and provide a wide experience for improving QoL. It will be conducted based on exercise (Tai Chi) for maintaining cognitive function and games for cognitive function. Also, web-based cognitive training developed by Dementia Center in Chungbuk Metropolitan in Korea will be used, and psychomotor therapy, therapeutic arts and crafts activities, and reminiscence therapy will be included. Psychomotor therapy is a treatment within the middle of sensorimotor therapy and psychotherapy and allows older adults to build positive relationships with their body with a comfortable experience of body. Reminiscence therapy helps older adults maintain cognitive function by evoking memories. Therapeutic arts and crafts activities aim to improve cognitive and hand function and prevent muscle degeneration (both small- and large-muscle groups). Specifically, art therapy and horticultural therapy can be performed. Each program will be performed by experts in the field.

(2) Caregiver program: Caregiver will be provided with personal psychological counseling and social services that share information about insurance expenses and vouchers. To improve understanding of dementia, drug and nutrition education will be conducted. Although it may change depending on circumstances, both patient and caregiver will participate in Tai Chi and psychomotor programs so that they can perform the programs at home.
(3) Remark: This program may change depending on opinions and feedback from the patient, caregiver, and experts every week.

##### 2.3.2.2. Program application schedule

Integrated services of Western and Korean medicine will be provided at the Departments of Neurology and Korean Neuropsychiatry in the medical institute, where the study is being conducted. Integrative service will take place in a safe place of the hospital (study institute), and the programs will be conducted 2 times a week (8 sessions in total) in the presence of medical staff. A different researcher will be assigned to each patient and caregiver, and the program will take about a maximum of 100 minutes for each session.

The program is described in detail in Table 1.

**Table 1:** Integrative medical service programs for the patient with dementia and his/her caregiver.

Session		Dementia program: Quality of life improvement						Time
**1**	P	Medical care (Western medicine↔Korean medicine)	Program explanation and completing the questionnaire (50 min)			BM (5 min)	Reminiscence therapy, feedback and evaluation (15 min)	≤100 min
	C	Program explanation and completing the questionnaire (40 min) caregiver full battery psychotherapy				BM (5 min)	Feedback and evaluation (5 min)	
**2**	P	Medical care (Western medicine↔Korean medicine)	BM (5 min)	Therapeutic arts and crafts activity (art therapy) (50 min)		BM (5 min)	Reminiscence therapy, feedback and evaluation (15 min)	≤100 min
	C	Caregiver’s psychology test interpretation (50 min)				BM (5 min)	Feedback and evaluation (15 min)	
**3**	P	Medical care (Western medicine↔Korean medicine)	BM (5 min)	Therapeutic arts and crafts activity (horticultural therapy) (50 min)		BM (5 min)	Reminiscence therapy, feedback and evaluation (15 min)	≤100 min
	C	Caregiver psychology consultation (50 min)				BM (5 min)	Feedback and evaluation (15 min)	
**4**	P	Medical care (Western medicine↔Korean medicine)	BM (5 min)	Exercise for maintaining cognitive function (Tai Chi) (40 min)	Cognitive activity program (10 min)	BM (5 min)	Reminiscence therapy, feedback and evaluation (15 min)	≤100 min
	C	Caregiver psychology consultation (15 min)		Exercise for maintaining cognitive function (Tai Chi) (40 min)		BM (5 min)	Feedback and evaluation (15 min)	
**5**	P	Medical care (Western medicine↔Korean medicine)	BM (5 min)	Psychomotor therapy for maintaining cognitive function (60 min)		BM (5 min)	Reminiscence therapy, feedback and evaluation (15 min)	≤100 min
	C	Caregiver psychology consultation (50 min)				BM (5 min)	Feedback and evaluation (15 min)	
**6**	P	Medical care (Western medicine↔Korean medicine)	BM (5 min)	Exercise for maintaining cognitive function (Tai Chi) (40 min)	Cognitive activity program (10 min)	BM (5 min)	Reminiscence therapy, feedback and evaluation (15 min)	≤100 min
	C	Caregiver psychology consultation (15 min)		Exercise for maintaining cognitive function (Tai Chi) (40 min)		BM (5 min)	Feedback and evaluation (15 min)	
**7**	P	Medical care (Western medicine↔Korean medicine)	BM (5 min)	Psychomotor therapy for maintaining cognitive function (60 min)		BM (5 min)	Reminiscence therapy, feedback and evaluation (15 min)	≤100 min
	C	Caregiver psychology consultation (50 min)				BM (5 min)	Feedback and evaluation (15 min)	
**8**	P	Medical care (Western medicine↔Korean medicine)	BM (5 min)	Cognitive activity program (5 min)	Completing questionnaire (40 min)	BM (5 min)	Reminiscence therapy, feedback and evaluation (15 min)	≤100 min
	C	Caregiver counseling (15 min)			Completing questionnaire (40 min)	BM (5 min)	Feedback and evaluation (15 min)	

BM = breathing meditation, C = caregiver, P = patient.

### 2.4. Evaluation

#### 2.4.1. Observed items.

1) Patient and caregiver’s demographic information:

1) Basic information about the patient.

- Completed by the patient: Sex, date of birth, marital status, education level, primary caregiver, duration of disease.
- Completed by the caregiver: Name, sex, date of birth, contact information, address, current possession of care, use of mobile phone, education level, years of education, illiterate, past occupation, current occupation, patient’s lifestyle (drinking alcohol, smoking, family history), medical information about the patient (vision, hearing, physical illness, and drugs currently taking), physical exercise (activity), brain activity, social activity, and brief dietary assessment table.

2) Basic information about the caregiver (a primary caregiver who provides direct care to the patient).

- Name, sex, age, relationship with the patient, contact information, living with patient, address, time spent to take care of the patient, average income, occupation, diseases, cost of hospitalization, burden of caregiver, rehabilitation, and insurance related to medical care for cognitive impairment.

2) Assessment of dementia and QoL in patients:

1) Cognitive assessment (MMSE, Clinical Dementia Rating (CDR), Global Deterioration Scale [GDS], and Montreal Cognitive Assessment-Korean [MoCA-K])

①MMSE^[12]^

- MMSE is a brief screening tool developed by Folstein et al in 1975. The maximum score is 30, and it takes about 5 to 15 minutes. Specifically, it has the advantages of assessing changes over time by repeatedly measuring disease progression due to its low learning effects.^[12]^ Generally, a score of 23 is the accepted cutoff point, and it indicates the presence of cognitive impairment. In epidemiological studies, an MMSE score of 24 to 30 is considered normal, while 18 to 23 is MCI and 0 to 17 is severe cognitive impairment.

②CDR scale

- The CDR is a tool for evaluating the severity of dementia. The CDR scale is a 0 to 3 point numeric scale derived from the rating of each domain and total CDR, but the expanded CDR version with a 0 to 5 numeric point scale was developed and divided the grade of severe dementia into CDR-3, 4, and 5.^[13]^ The CDR measures 6 six domains: memory, orientation, judgment and problem-solving, community affairs, home and hobbies, and personal care. Each domain can be rated on a 5-point scale (0, 0.5, 1, 2, 3, 4, and 5). The doctor rates each domain after assessing the functions of the 6 domains through close interviews with the patient and caregiver.

③GDS

- The GDS,^[14]^ which is widely used in Europe, assesses the severity of degenerative dementia. A standardized study was conducted using the Korean version of GDS.^[15]^ GDS 1 indicates clinically normal condition with no cognitive decline, GDS indicates subjective memory deficits, and GDS 3 indicates mild cognitive decline. Some patients with mild dementia are included in GDS 3. GDS ≥4 is definitely dementia. GDS 4 indicates mild dementia, GDS 5 indicates moderate dementia, while GDS 6 and 7 indicate severe dementia.^[15–17]^

④MoCA-K

- The MoCA-K is a screening tool developed to select individuals with MCI, which shows normal manifestations from the MMSE.^[18–20]^
- The MoCA-K is composed of short-term memory, visuospatial abilities, executive functions, attention, concentration and working memory, language, and orientation, and the total score is 30 points. It takes about 10 minutes, and a score of ≥23 is considered normal.

For those who are not able to read or write or are not proficient in reading or writing, the MoCA-K is not recommended.

(2) Assessment of activities of daily living (Seoul-Instrumental Activities of Daily Living [S-IADL], Korean version of Barthel Activities of Daily Living Index [K-BADL])

①S-IADL

- Items of the S-IADL^[21]^ were adapted to Korean cultural characteristics and standardized. It is composed of 15 items with a 3-point numeric scale. The total scores of the S-IADL range from 0 to 45, with higher scores indicating poor performance in activities of daily living. This tool assesses IADL by dividing it into the performance of IADL at the present time and potential performance. “The performance of IADL at the present time” assesses whether a patient can currently perform IADL independently. Meanwhile, “potential performance” assesses whether a patient can perform IADL taking the patient’s potential ability into consideration, although the patient does not currently perform IADL independently. This tool showed that a cutoff score for healthy people and dementia patients was 7.5 points (if ≥8, an indicative of dementia).

②K-BADL

- K-BADL was used to measure basic activities of daily living. Total scores range from 0 to 20, and a score of 11 to 15 is considered moderate dependency while ≤10 points is considered fully dependency.^[22]^

(3) Assessment of abnormal behavior (Neuropsychiatric Inventory Questionnaire [NPI-Q])

NPI-Q, a brief questionnaire form of the neuropsychiatric inventory, has been designed to be used in the medical field. Each of the 12 items was well correlated with the NPI (*R* = 0.71–0.93). It takes ≥15 minutes to complete the NPI, while it takes ≤5 minutes to complete the NPI-Q.^[23]^ In this study, the Korean version of NPI-Q will be used.^[24]^

(4) Assessment of depressive symptoms in older adults (Korean version of the short form of Geriatric Depression Scale )

The Korean version of the short form of Geriatric Depression Scale, which was revised by Cho et al,^[25]^ was used to measure depressive symptoms in older adults, and it is composed of 15 items. A score of 5 indicates depression suspect.

(5) Core Seven-Emotions Inventory-Short Form^[26]^

The Core Seven-Emotions Inventory-Short Form is composed of 28 items including 4 items for joy, anger, thought, depression, sorrow, fear, and fright, respectively, each with a 5-point Likert scale. This scale is based on the T-score (mean score of 50 and standard deviation of 10), and high scores in anger, thought, depression, sorrow, fear, and fright, except for joy, indicate a high-risk group. In terms of specific cutoff points, a score between 55 and 60 indicates a caution group, 61 and 65 indicates a risk group, and ≥66 indicates a high-risk group. Conversely, for joy, a low score indicates a risk group. A score between 40 and 45 indicates a caution group, 35 and 39 indicates a risk group, and ≤34 indicates a high-risk group.

However, feeling cards can be used for individuals with advanced dementia. In terms of emotional assessment in older adults with dementia, individuals with a score of MMSE-K ≥10 points have been reported not to show difficulty in expressing their internal state despite cognitive impairment.

(6) Geriatric QoL-Dementia

The Geriatric QoL-Dementia has a total of 15 items, with 13 items on physical health, psychological health, social relations, and environment, 1 item on general health, and 1 item on life satisfaction. Each item is rated on a 3-point Likert scale. The total score is calculated by adding a score of each item and ranges from 15 to 60. The total score is interpreted by converting it into a norm score (T-score) that considers sex and age. A high norm score means that a patient has a high subjective satisfaction with QoL. A norm score of ≤35T refers to low QoL.^[24]^

3) Assessment of caregiver’s QoL

(1) Primary caregiver burden inventory

To adapt the Caregiver Burden Inventory developed by Novak and Guest (1989),^[27]^ Jang^[28]^ translated and modified this tool. This tool comprises 29 questions divided into 6 dimensions: time-dependence (1–5), developmental and achievement (6–10), physical (11–14), social (15–19), emotional (20–24), and financial (25–29) dimensions. Each item is rated on a scale from 1 (not at all descriptive) to 5 (very descriptive). The total score ranges from 29 to 145, where higher scores indicate a greater burden.

(2) Measurement of QoL in caregiver (EuroQoL-5 Dimension 5-Level [EQ-5D-5L] and EuroQol Visual Analogue Scale [EQ-VAS])

① Measurement of health-related QoL (HRQoL) (EQ-5D-5L)

EQ-5D was developed by the EuroQol Group, initially established in 1987 and being consecutively developed. Korean EQ-5D is a tool for measuring HRQoL by converting it into a utility score. It is a multidimensional preference-based HRQoL measurement. The EuroQol Group developed a new EQ-5D version to supplement the disadvantages of EQ-5D-3L, by increasing the number of responses from 3 to 5. The EQ-5D-3L could give 243 health states. However, since the EQ-5D-5L can give 3125 (=55) health states, it had a smaller ceiling effect than EQ-5D-3La, and reliability and sensitivity have increased.^[29]^

② General health status score (EQ-VAS)^[30]^

The EQ-VAS is one of the rating scales and is a vertical 20 cm visual analog scale. Each item is rated on a scale from 0 to 100 with each endpoint labeled “the best health you can imagine (100)” and “the worst health you can imagine (0).” Scores from the rating scales elicit the order of health results clearly and information about the degree of preference.

(3) Oxford Happiness Scale

To measure happiness levels, the Oxford Happiness Scale, which was developed by Hills and Argyle (2002),^[31]^ was used after it was translated by Choi and Lee (2004).^[32]^ This tool measures the level of self-control, positive emotion, and self-esteem with 29 items. Each item is rated on a scale from 1 to 6 with each endpoint labeled “strongly disagree (1 point)” to “strongly agree (6 points),” and we reversed the score of the negatively worded questions. Higher scores indicate a higher level of happiness.

4) Evaluation of feedback at each session

(1) Projective assessment of patient and caregiver after the end of the session (Mentalizing the Room of Mind [MRM])

MRM is one of the Mindfulness and Loving Beingness psychotherapy techniques that enable observing the mind that the patient is not aware of it more objectively by visualizing and embodying the state of the mind at the moment.^[33]^ This tool helps individuals to objectify their minds in a mutual relationship and counselees (participants) to organize their vague feelings one by one through introspection. This is a useful instrument that enables assessing it and observing progression and a clinically highly utilized tool.

(2) Report on the details of feedback from each session

After the end of each session, each investigator who explained the program to participants (a caregiver and patient) will collect and record feedback. After that, the Principal Investigator, each investigator who collected feedback from the caregiver and the patient, respectively, and experts will gather to give their feedback on the program at each session. Specifically, general feedback will be on whether there have been any insufficient parts and areas for improvement, and cost parts when future research on this program is conducted in a medical institute. Feedback will be reflected in the program at the subsequent session to improve the program.

#### 2.4.2. Evaluation schedule.

(1) Participant evaluation

Assessment items will be collected 2 times from the study participants, before applying the first program and after Session 8. Observation assessment for the program will be conducted by collecting MRM and feedback for each session through an interview at each session. The study schedule is described in Table 2.

**Table 2:** Study schedule table.

Period			Screening (consent, enrollment)	1st investigation (1 wk)	2nd investigation (8 wk)
Written informed consent form completion			•		
Inclusion/Exclusion criteria determination			•		
Taking sociodemographic information and medical history				•	
Utilization of long-term care service				•	•
Investigation of underlying disease	Diagnosis of dementia in Oriental medicine		•	•	
	EQ-5D, EQ-VAS		•	•	•
Patient clinical-related assessment	MMSE		•		•
	CDR			•	•
	GDS			•	•
	MoCA-K			•	•
	SGDS-K			•	•
	GQOL-D			•	•
	CSEI-s			•	•
Caregiver questionnaire assessment	Activities of patient’s daily living assessment	Barthel-ADL		•	•
		S-IADL		•	•
	Patient’s abnormal behavior assessment	NPI-Q		•	•
	Caregiver evaluation	Quality of life (EQ-5D, EQ-VAS)		•	•
		Measurement of caregiver burden		•	•
		CSEI-s		•	•
		Happiness scale		•	•

MRM and feedback will be collected every session.

CDR = Clinical Dementia Rating, CSEI-s = Core Seven-Emotions Inventory-Short Form, EQ-5D = EuroQoL-5 Dimension 5-Level, EQ-VAS = EuroQol Visual Analogue Scale, GDS = Global Deterioration Scale, GQOL-D = Geriatric Quality of Life-Dementia, MMSE = mini-mental state examination, MRM = Mentalizing the Room of Mind, MoCA-K = Montreal Cognitive Assessment-Korean, NPI-Q = Neuropsychiatric Inventory Questionnaire, SGDS-K = Korean version of the short form of Geriatric Depression Scale, S-IADL = Seoul-Instrumental Activities of Daily Living.

(2) Investigator assessment

After the end of each session, the Principal Investigator, each investigator who collected feedback from the caregiver and the patient, respectively, and experts will gather to give their feedback on the program at each session.

### 2.5. Analysis method

Data will be divided into quantitative and qualitative data for analysis.

1) Quantitative analysis: Questionnaire responses from patients and caregivers about QoL improvement in patients with dementia and clinical characteristics of dementia will be analyzed. In terms of statistical analysis, the paired samples *t*-test, among the inferential statistics, will be conducted for each questionnaire before and after the program (before the first session and after the end of session 8), and exploratory data analysis will be conducted.
2) Qualitative analysis: Feedback from the patient and caregiver on the program and MRM, the projective assessment of emotional expression, will be qualitatively analyzed after applying the program at each session.

### 2.6. Monitoring

Apart from the research team, a research nurse and expert monitoring team will be established to conduct ongoing supervision to collect, assess, report, and manage reported negative events and other unintended effects of test intervention or experimental behavior. In addition, all of these actions will be independent of interest.

### 2.7. Ethical consideration

This study protocol was submitted to the IRB of the Wonwkang University Sanbon hospital and was approved on July 8, 2022 (IRB No. WMCSB 202206-52). Since this is an observational study, we do not believe that there is enormous additional risk in addition to the risk from general healthcare practice. However, since an interview may induce mental and psychological risks, a measure should be taken to immediately connect the patient and caregiver to their counselor and an expert specializing in Korean Neuropsychiatry Medicine.

The program carried out in this study takes approximately 100 minutes, which may induce physical or mental exhaustion. Thus, we will take 10-minute rest breaks after carrying out the program for 1 hour. Also, the participants can have rest breaks at any time during the program if they want to.

Information and data obtained from the participants will be used after being protected reliably so that their identities will not be directly identified. If the results of this study are published, the personal information of the participants will not be included in any of the study results.

All data collected and obtained during the study will not be used for purposes other than for completing the clinical study report. Participants’ data will be locked in a cabinet in the Principal Investigator’s office to protect against loss and theft. After the end of the study, the data will be stored in a file cabinet with lock in the Principal Investigator’s office for 3 years to protect against loss and theft, and then be discarded.

## 3. Discussion

The previous study on the integrative medical service model discussed that cognitive rehabilitation and exercise should be considered for patients with cognitive decline to maintain cognitive function at home.^[3]^ According to this discussion, the integrative medical service program for patients with dementia was developed based on cognitive rehabilitation and exercise. Also, the caregiving burden is expected to be reduced while QoL is expected to be improved in the caregiver by providing psychosocial service to caregiver of the patient with dementia who has a high caregiving burden with low QoL. ^[34]^ Since this is a single-case protocol, we may not be able to collect various opinions. However, we will attempt to gather depth opinions by receiving sufficient feedback from the patients and caregivers at each session. Moreover, we can conduct the study by modifying the program based on feedback obtained at each session unlike a multi-case study which cannot conduct the program flexibly. Accordingly, we can identify which program is the most satisfactory and helpful so that the patients and caregivers can improve their QoL from their point of view. We expect that the integrative medical service model can be revised to the optimized patient-based model. A clinical pathway development study, which applies the revised model to medical institutes, can be conducted in the future.

## Author contributions

**Conceptualization:** Moon Joo Cheong, Do-Eun Lee, Hyung Won Kang.

**Data curation:** Moon Joo Cheong, Do-Eun Lee, Hyung Won Kang.

**Formal analysis:** Moon Joo Cheong.

**Funding acquisition:** Hyung Won Kang.

**Investigation:** Moon Joo Cheong.

**Methodology:** Moon Joo Cheong.

**Project administration:** Moon Joo Cheong.

**Supervision:** Hyung Won Kang.

**Writing – original draft:** Moon Joo Cheong.

**Writing – review & editing:** Moon Joo Cheong, Do-Eun Lee, Jeesu Kim, Il-Hong Son, Sung Chul Kim, Hyung Won Kang.
